# Revision of the Neotropical water scavenger beetle genus *Quadriops* Hansen, 1999 (Coleoptera, Hydrophilidae, Acidocerinae)

**DOI:** 10.3897/zookeys.705.19815

**Published:** 2017-10-03

**Authors:** Jennifer C. Girón, Andrew Edward Z. Short

**Affiliations:** 1 Department of Ecology & Evolutionary Biology, and Division of Entomology, Biodiversity Institute, University of Kansas, Lawrence, KS 66045, USA

**Keywords:** terrestrial aquatic beetles, new species, taxonomy

## Abstract

The genus *Quadriops* Hansen, 1999 is revised and redescribed. The genus is found to contain six species, including two that are here described as new: *Quadriops clusia* **sp. n.** (Brazil, Guyana, Suriname) and *Q. acroreius* **sp. n.** (Suriname, French Guiana). Two species are found to be junior subjective synonyms of *Q. depressus* Hansen, 1999: *Q. amazonensis* García, 2000, **syn. n.** and *Q. politus* Hansen, 1999, **syn. n.** The male of *Q. similaris* Hansen, 1999 is described for the first time. New records are provided for *Q. dentatus* Hansen, 1999, *Q. reticulatus* Hansen, 1999, and *Q. similaris*. All species are described and illustrated in detail. Most species are confirmed as having a terrestrial way of life, with several species being found in rotten fruits, sap flows, and dead wood. Furthermore, we discuss ecological trends of the species given their collecting information.

## Abstract (es)

El género *Quadriops* Hansen, 1999 es revisado y redescrito. El género contiene seis especies, incluyendo dos que se describen aquí como nuevas: *Quadriops clusia* **sp. n.** (Brasil, Guyana, Surinam) y *Q. acroreius* **sp. n.** (Surinam, Guyana Francesa). Dos especies se sinonimizan con *Q. depressus* Hansen, 1999: *Q. amazonensis* García, 2000, **syn. n.** y *Q. politus* Hansen, 1999, **syn. n.** El macho de *Q. similaris* Hansen, 1999 se describe por primera vez. Se proveen nuevos registros para *Q. dentatus* Hansen, 1999, *Q. reticulatus* Hansen, 1999, y *Q. similaris*. Todas las especies son descritas e ilustradas en detalle. La mayoría de las especies presentan un modo de vida terrestre, con varias especies encontradas en frutos podridos, flujos de savia y madera muerta. Además, se discuten tendencias ecológicas de las especies dada su información de colecta.

## Introduction

The water scavenger beetle genus *Quadriops* Hansen, 1999 is endemic to the Neotropical region, with a known distribution from as far north as Costa Rica to as far south as Amazonian Peru (Hansen 1999). When the genus was originally described, Hansen (1999) placed *Quadriops* in the subtribe Acidocerina of the tribe Hydrophilini (*sensu* Hansen 1991), which contemporarily mostly constitutes the subfamily Acidocerinae (Short and Fikáček 2013).

Species of *Quadriops* can be easily recognized by their small size (ca. 2 mm), completely divided eyes, short and stout maxillary palps, mostly glabrous posterior femora, and the rounded apex (as opposed to truncate or emarginate) of the fifth abdominal ventrite. In general terms, and as happens with some other Neotropical acidocerines (e.g., *Globulosi*s García, 2001), the external morphology is highly homogeneous among species. However, the distribution of the elytral punctures constitutes a useful character to recognize species groups.

Previous species descriptions were based on one or a few specimens, all but one of them collected by flight intercept traps. According to Hansen (1999), most diagnostic features that separate species involved the presence, density, and location of microsculpture or reticulation on the clypeus, head and pronotum. Furthermore, by examining the illustrations of the aedeagus provided by Hansen (1999) and García (2000), similarities are evident among the described males.

Recent fieldwork in northern South America has significantly expanded our knowledge of *Quadriops*. This has included increasing the number of known specimens by almost 100 fold, expanding the range of some species, as well as revealing new species and habits of the beetles unknown until now. Based on all the gathered material, here we redescribe the genus and the previously known species, based on morphological characters of the adults. We synonymize *Q. amazonensis* García, 2000 and *Q. politus* Hansen, 1999 with *Q. depressus* Hansen, 1999, based on external morphology as well as on characters of the aedeagus. Additionally, we describe two new species: *Q. acroreius* sp. n. from French Guiana and Suriname, and *Q. clusia* sp. n. from Guyana, Suriname, and Brazil, which has been collected on the rotten fruits of *Clusia* trees. We also discuss the ecology and distribution of the species.

## Materials and methods

Depositories of examined material:

**CBDG** Center for Biological Diversity, University of Guyana, Georgetown

**CMNC** Canadian Museum of Nature, Ottawa, Canada (R. Anderson)

**INBio** Instituto Nacional de Biodiversidad, Santo Domingo, Costa Rica.

**MALUZ** Museo de Artrópodos de la Universidad del Zulia, Maracaibo, Venezuela (J. Camacho, M. García)

**MIZA** Museo del Instituto de Zoología Agrícola, Maracay, Venezuela (L. Joly);

**NHMUK** United Kingdom, London, The Natural History Museum [formerly British Museum (Natural History BMNH)]

**NZCS** National Zoological Collection of Suriname, Paramaribo (P. Ouboter, V. Kadosoe)

**SEMC** Snow Entomological Collection, University of Kansas, Lawrence, KS (A. Short)

**USNM** U.S. National Museum of Natural History, Smithsonian Institution, Washington, DC (C. Micheli).

**Morphological methods.** Specimens were examined using Olympus SZX7 and SZX16 stereo microscopes (magnifications: 0.8–5.6× with DF PLAPO 1–4× objective lens and 20× eyepieces; 0.7–11.5×, with SDF PLAPO1×PF objective lens and 10× eyepieces, respectively). Genitalia dissections were prepared, in part, by the protocols described by Minoshima et al. (2015), by heating the structures at 60°C in a solution of 10% KOH during 60 minutes. Previous to the KOH treatment, the entire abdomen was removed from the specimen and opened along one side. Afterwards, structures were submerged in glacial acetic acid for 15 minutes, and then rinsed with distilled water. Dissections were performed by placing the cleared parts on a microscope slide with a drop of glycerin. The aedeagi of male holotypes designated by Hansen, originally mounted in Euparal on cards pinned under the specimens, were dismounted by placing the card in 70% alcohol in a water bath (~60°C, 15-20 min) and then observed on a microscope slide with a drop of glycerin.

Images of internal structures were produced by stacking images taken through an Olympus DP72 camera attached to an Olympus BX51 microscope to 200× magnification. Habitus photographs were taken with a Visionary Digital imaging system, using a Canon MP-E 65mm f/2.8 1–5X Macro Lens mounted on a Canon EOS 6D camera body. All final images were created by stacking multiple individual photographs from different focal planes using the software Zerene Stacker. Scanning electron micrographs were taken by using a FEI Versa 3D Dual Beam Scanning Electron Microscope. Specimens were mounted on carbon tape and coated in gold.

Descriptive sequence and morphological terminology largely follows Hansen (1991) except for the use of meso- and metaventrite instead of meso- and metasternum (see Lawrence and Ślipiński 2013). Terms for the ventral surface of head follow Komarek (2004). Terminology for the metafurca follows Velázquez de Castro (1998). Wing venation follows Lawrence and Ślipiński (2013). The generic description has been modified from Hansen (1999).

In the examined material section of the descriptions, the sex of the specimens is indicated only for those in which the genitalia was exposed. For the remainder specimens the sex was not determined.

## Results

### List of species

*Quadriops acroreius* sp. n. Suriname, French Guiana

*Quadriops clusia* sp. n. Guyana, Suriname, Brazil

*Quadriops dentatus* Hansen, 1999 Venezuela (Bolivar), French Guiana, Suriname

*Quadriops depressus* Hansen, 1999 Peru, Ecuador, Venezuela (Amazonas)

*Q. amazonensis* García, 2000, syn. n.

*Q. politus* Hansen, 1999, syn. n.

*Quadriops reticulatus* Hansen, 1999 Costa Rica, Panama

*Quadriops similaris* Hansen, 1999 Venezuela (Bolivar), Guyana, Suriname, French Guiana

### Characters of taxonomic portance

**Figure 1.**
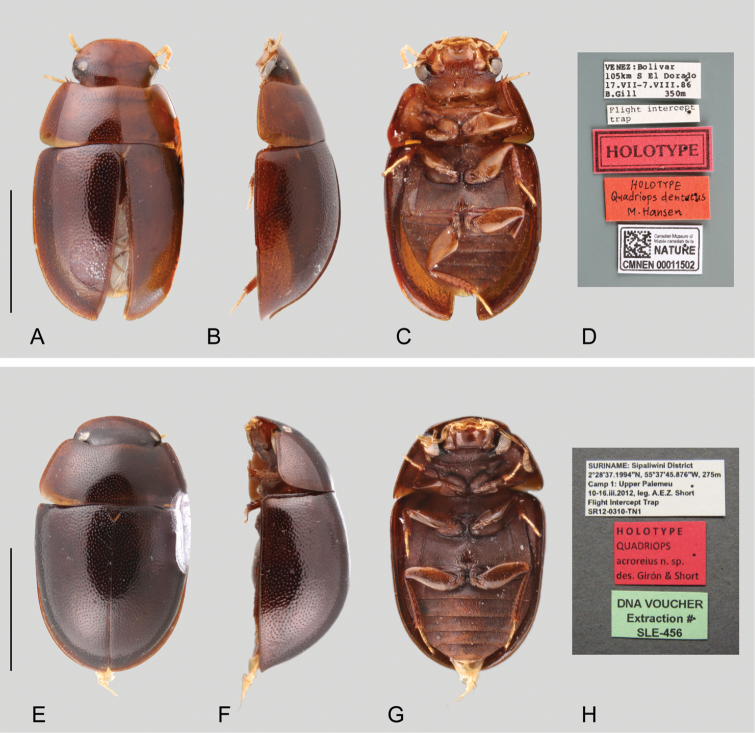
Habitus and labels of *Quadriops* spp.: *Q. dentatus* (holotype): **A** dorsal view **B** lateral view **C** ventral view **D** labels; *Q. acroreius* sp. n. (holotype): **E** dorsal view **F** lateral view **G** ventral view **H** labels. Scale bar 1 mm.

**Figure 2.**
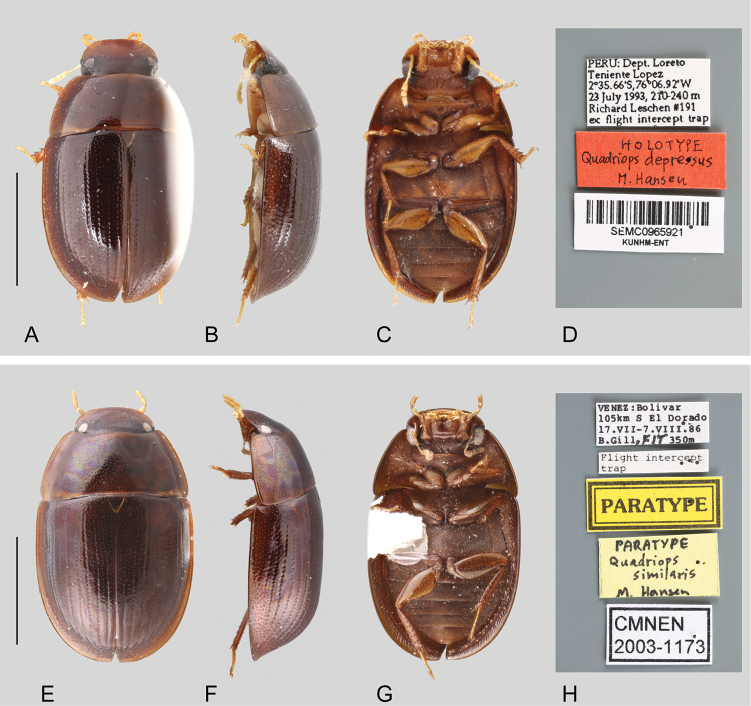
Habitus and labels of *Quadriops* spp.: *Q. depressus* (holotype): **A** dorsal view **B** lateral view **C** ventral view **D** labels; *Q. similaris* (paratype): **E** dorsal view **F** lateral view **G** ventral view **H** labels. Scale bar 1 mm.

**Figure 3.**
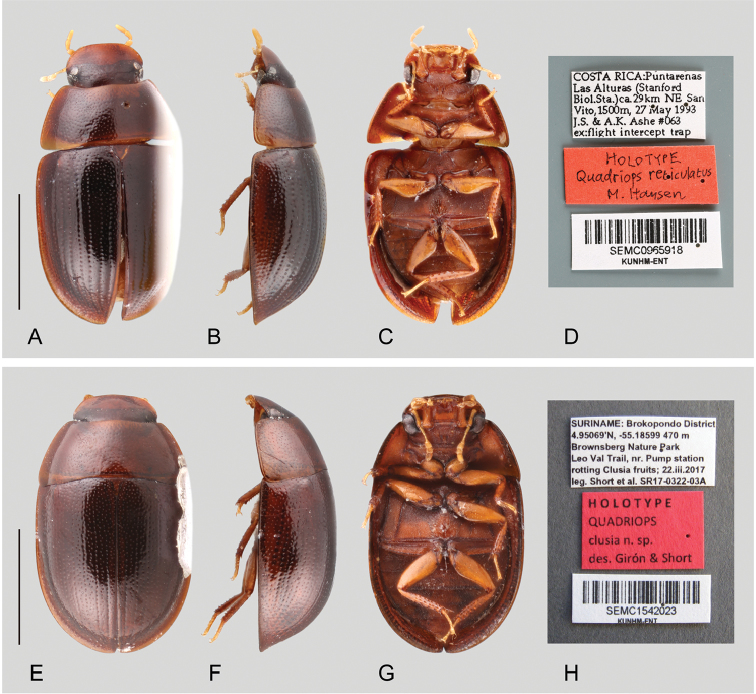
Habitus and labels of *Quadriops* spp.: *Q. reticulatus* (holotype): **A** dorsal view **B** lateral view **C** ventral view **D** labels; *Q. clusia* sp. n.: **E** dorsal view **F** lateral view **G** ventral view **H** holotype labels. Scale bar 1 mm.

For the most part, species of *Quadriops* are externally homogeneous (at least within the species groups). We also observed very low intraspecific variation, even across large series (hundreds) of specimens. Variation was found mainly in the following characters:

**Body shape in lateral view.** Though the shape of the body in lateral view might be described as subhemispherical, there is interspecific variation in the degree of convexity. The outline of some species can be described as uniformly convex: *Q. dentatus*, *Q. acroreius*, *Q. reticulatus* and *Q. clusia* (see Figs 1B, F, 3B, F respectively), whereas *Q. depressus* and *Q. similaris* are more dorsoventrally flattened (Figs 2B, F).

**Coloration.** Body coloration, which tends to be uniform across most regions of the beetle, does not represent a diagnostic feature for separating *Quadriops* species. While coloration in specimens examined ranges from yellowish to reddish to dark brown, this is mostly attributable to intraspecific variation as well as varying degrees of sclerotization. Appendages and the ventral side of the beetles tend to be slightly paler than the dorsal surface of the body.

**Microsculpture.** One of the main characters used by Hansen (1999) to differentiate species was the presence, density and extension of microreticulations on the head, frons, and pronotum. Even if it might be useful in recognizing particular species (e.g. *Q. acroreius* vs. *Q. dentatus*), by looking at series of specimens, it is a variable character that should not be considered exclusively diagnostic.

**Elytra.** Two main groups of species can be distinguished according to the distribution of the ground and serial punctures of the elytra: those in which the punctures are randomly and uniformly distributed over the surface (*Q. acroreius* and *Q. dentatus*, see Fig. 1), and those in which the punctures are serially arranged, forming well defined longitudinal striae (see Figs 2 and 3). With the exception of *Q. clusia*, in striate species the punctures along the striae are clearly larger than those on the interstria (in *Q. clusia* all elytral punctures are similarly large; see Fig. 6). In addition, the elytral punctures can be simple as in *Q. clusia* (Fig. 6A) or possess microincisions that radiate from the margins of the puncture (ramified punctures) as in *Q. reticulatus* (Fig. 6B).

**Mesoventrite.** In *Quadriops*, the mesoventrite is broadly elevated posteriorly. The wide elevation usually has a transverse ridge, which varies in shape and sharpness. In the known species with irregularly distributed elytral punctures, the transverse ridge is strongly produced. It forms a blunt, vertical, median tooth in *Q. dentatus*, whereas in *Q. acroreius* it forms a wide, transverse, straight and blunt carina. As for species with elytral punctures aligned into striae, the transverse ridge can be simply curved, slightly angulate or bisinuate. For this group of species, the shape of the transverse ridge exhibits more intraspecific variation, and is not consistent within series of specimens.

**Aedeagus.** The general shape of the aedeagus and the length ratio between the basal piece and the median lobe + parameres is generally consistent within species. There is both inter- and intraspecific variation in the outer margins of the basal piece and the shape of the outer margins and apex of the parameres. The gonopore is positioned at the apex of the median lobe, but its shape exhibits intraspecific variation. The shape of the apices of both the median lobe and the parameres tend to be consistent within species. It is important to highlight that some differences observed in the aedeagi presented here may be a result of incomplete clearing, owed to positioning during the photographing process, and/or product of imperfect focus from the stacking process.

#### Quadriops

Taxon classificationAnimaliaColeopteraHydrophilidae

Hansen, 1999

Quadriops Hansen, 1999: 131.

##### Type species.

*Quadriops depressus* Hansen, 1999 by original designation.

##### Differential diagnosis.

Small to very small beetles, total body length 1.6–2.6 mm, width 1.1–1.6 mm. Color yellowish to reddish to dark brown. Body shape oval in dorsal view; subhemispherical and sometimes dorsally flattened in lateral view (see Figs 1–3). Frons lateral and posteriorly expanded, forming a canthus that completely divides the eyes in dorsal and ventral portions (e.g. Fig. 1B, 3B). Antennae with nine antennomeres (e.g. Fig. 2C). Maxillary palps curved inward, rather short and stout (e.g. Figs 1C, 2G). Elytra with punctures either irregularly distributed (Fig. 1) or forming ten well defined longitudinal rows, with additional elytral ground punctures along interstriae (Figs 2, 3); elytra without sutural striae, narrowly explanate anteriorly, explanation gradually broader towards apex (see Figs 1–3). Posterior elevation of mesoventrite, usually with a well-defined transverse ridge (except in *Q. dentatus* which possesses an acute tooth). Posterior femora glabrous with the exception of a few very scattered small setae. Fifth abdominal ventrite apically rounded and without stout setae (e.g. Fig. 1C).

##### Description.

**Figure 4.**
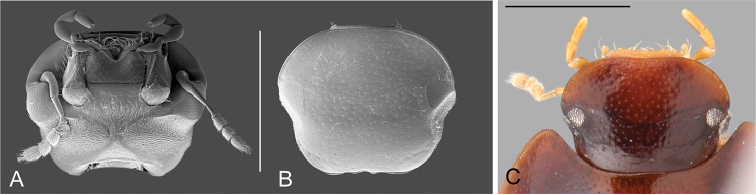
Head of *Q. reticulatus*: **A** SEM ventral view **B** SEM dorsal view **C** dorsal view. Scale bar: 0.5 mm.

**Figure 5.**
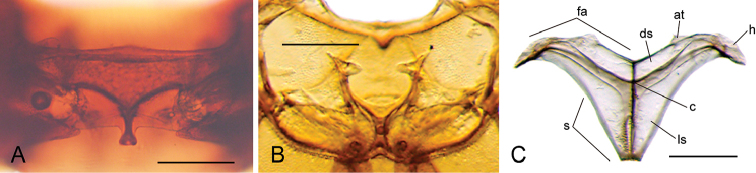
Thorax of *Q. reticulatus*: **A** prosternum ventral view **B** mesofurca dorsal view **C** metafurca posterior view: (at) anterior tendon, (c) crux, (ds) dorsal sheath, (fa) furcal arm, (h) hemiductus, (ls) lateral sheath, (s) stalk. Scale bar 0.2 mm.

**Figure 6.**
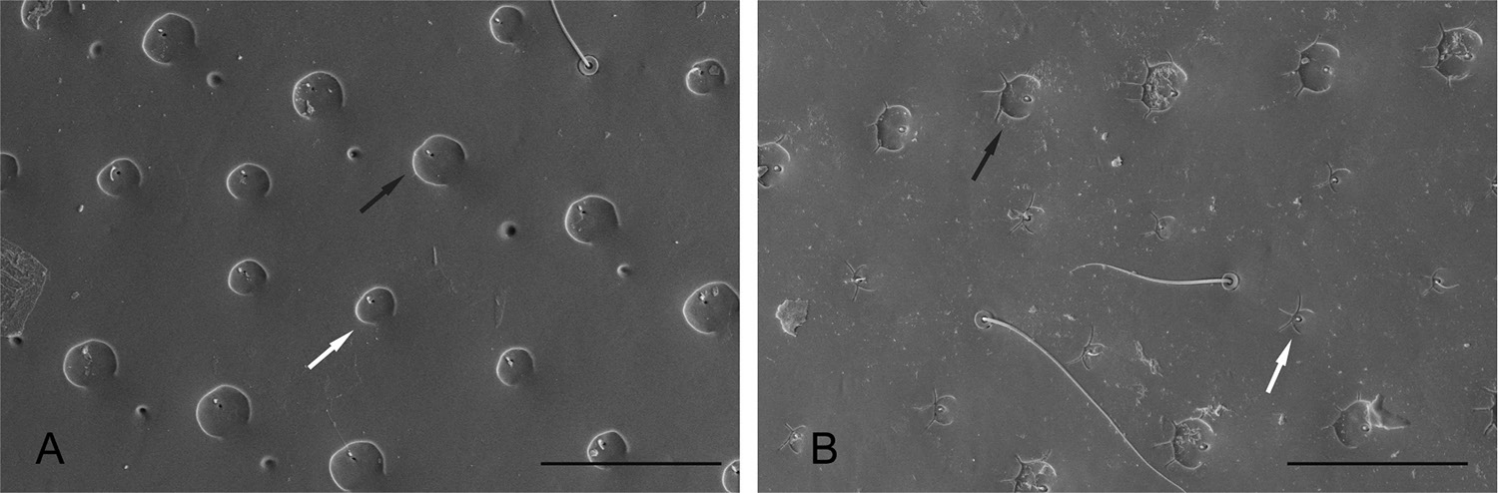
SEM of elytral punctures: **A** *Q. clusia* **B** *Q. reticulatus*. Scale bar: 50 µm. Black arrows point to serial punctures. White arrows point to interstrial punctures.

**Figure 7.**
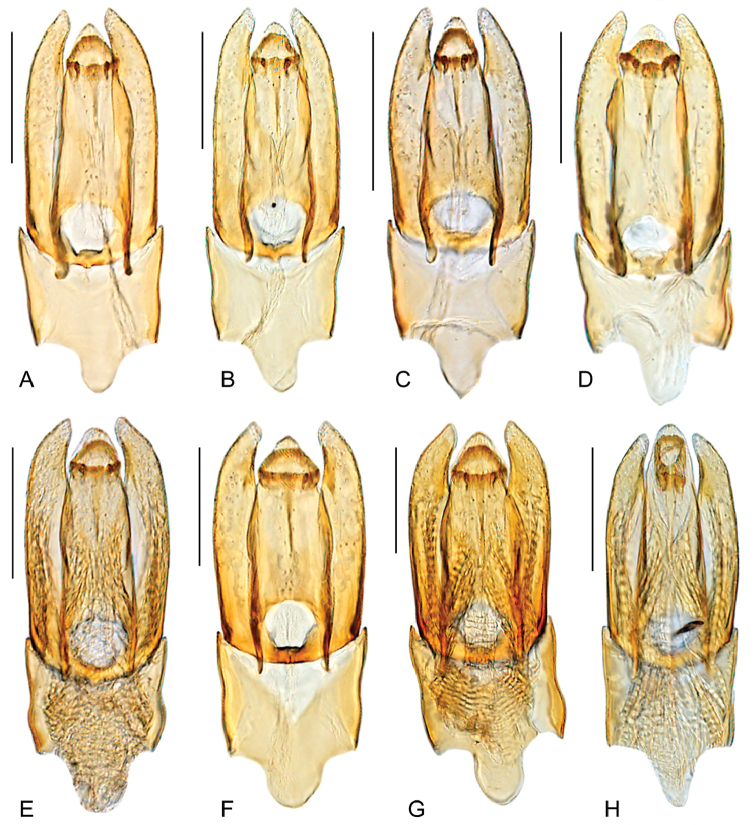
Aedeagus of *Quadriops* spp.: *Q. depressus*: **A** holotype **B** ‘*politus*’ holotype **C** ‘*amazonensis*’ holotype **D** VENEZUELA: Amazonas: Cerro de la Neblina; *Q. reticulatus*: **E** holotype **F** PANAMA: Chiriquí **G** COSTA RICA: Puntarenas [CR1ABF00 059] **H** COSTA RICA, Heredia [CR-11TN/16/016]; *Q. clusia* **I** BRAZIL: Manaus **J** GUYANA, Upper Potaro [GY14-0312-04A] **K** GUYANA, Ayanganna Airstrip [GY14-0317-01B]. Scale bar 0.1 mm.

**Figure 8.**
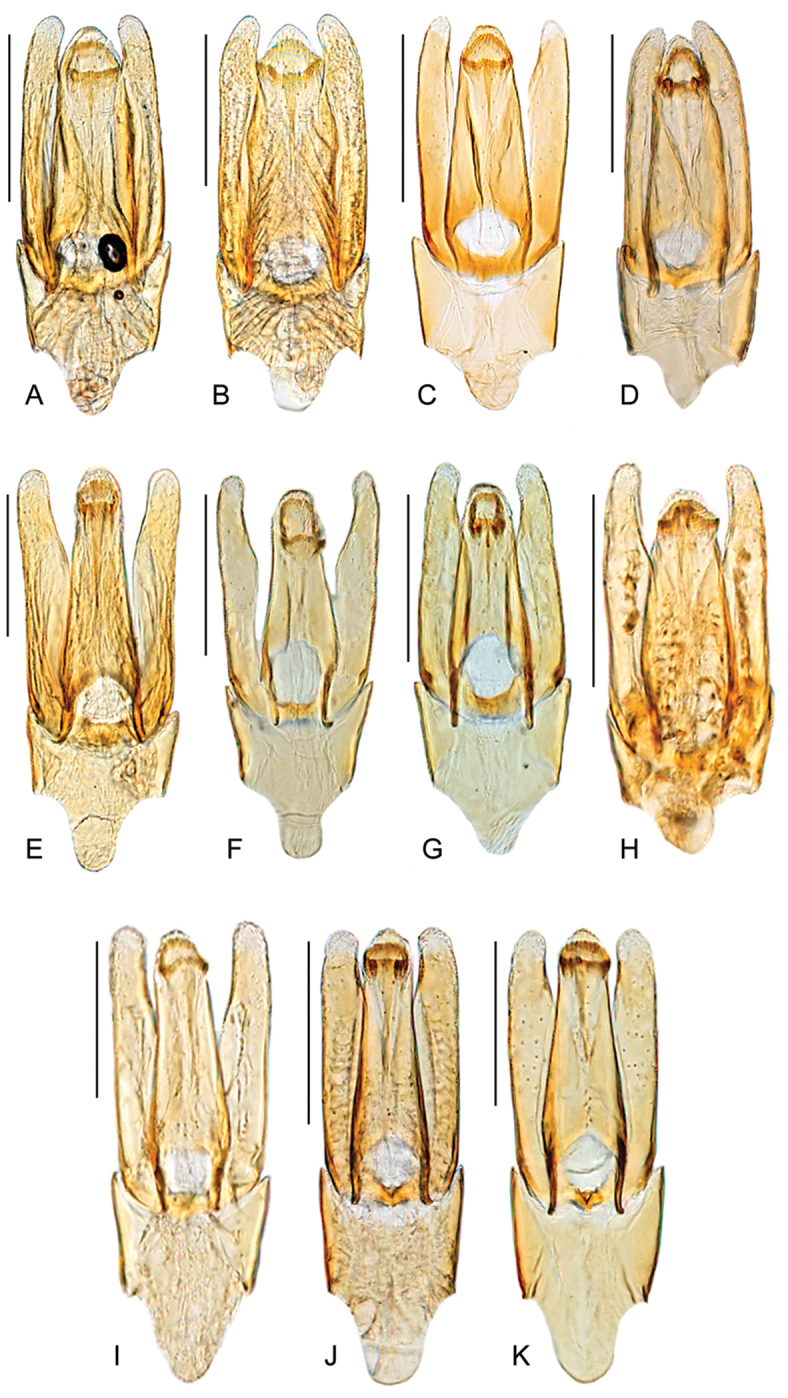
Aedeagus of *Quadriops similaris*: **A** SURINAME: Marowijne: Palumeu [SUR1F99 164] **B** GUYANA: Iwokrama Forest [GUY1BF01 005] **C** BRASIL: Manaus **D** SURINAME: Marowijne: Palumeu [SUR1F99 182] **E** FRENCH GUIANA: Roura [FG1AB97 027] **F** GUYANA: Iwokrama Forest [GUY1BF01 105] **G** FRENCH GUIANA: Cayenne: [FG1AB97 171] **H** SURINAME: Saramacca [SUR1F99 070]. Scale bar 0.1 mm.

Body broadly oval, weakly convex, with dorsum distinctly flattened in some species. **Head.** Eyes completely divided into dorsal and ventral faces by lateral canthus of the frons; dorsal face of eye tear-drop shaped, smaller in size relative to the ventral face. Antennae (see Fig. 4A) with 9 antennomeres, usually paler than general coloration of head; antennomere 1 reaching midpoint of ventral face of eye (reaching cardo-stipes joint), nearly 1.5-times longer than antennomere 2; antennomere 3 nearly as long as antennomeres 4–5 combined; antennomere 6 forming a rather small, but well differentiated cupule, antennomeres 7–9 similar in size, slightly flattened, forming a loosely articulated, pubescent club; setae at apex of antennomere 9 longer than general pubescence of club. Temporae forming a rather flat surface behind the eyes, densely covered by setae (hydrofuge pubescence, see Fig. 4B). Frons and clypeus (see Fig. 4A, C) with ground punctures uniformly distributed over the surface, accompanied by scattered seta-bearing systematic punctures; setae particularly noticeable on frons anterior to the eye, including lateral canthus, and behind frontoclypeal suture; surface between punctures ranging from smooth to finely reticulated, especially on anterior region of clypeus; anterior corners of clypeus widely rounded; anterior margin of clypeus usually emarginate medially, with distinct bead along entire margin. Labrum reduced, paler, rather short and wide, sometimes appearing deflexed and concealed by clypeus from above (see Fig. 4C); dorsal surface convex and finely reticulated; anterior margin mesally widely emarginate and bent inwards; lateral margins bearing a row of long setae. Maxilla (see Fig. 4A) usually with sparse setae on ventral surface of cardo and stipes, with a row of stiff decumbent spiniform setae along outer dorsal margin of palpifer; maxillary palps yellowish, shorter than antennae, and somewhat stout; palpomeres similar in size; palpomere 1 shorter than stipes, with inner margin straight, and outer margin distally strongly convex; palpomere 2 conical (narrower at base), with inner margin convex at base and outer margin widely convex; palpomere 3 digitiform, rather elongate (compared to 1 and 2), apically somewhat truncate; apex of palpomere 3 bearing sensilla. Mandibles with apex bifid (examined in *Q. clusia* and *Q. reticulatus*). Labial palps yellowish, nearly as long as mentum, dorsoventrally flattened; palpomere 2 with inner margin straight, and outer margin distally strongly convex, with a long seta on outer apical corner; palpomere 3 digitiform, usually shorter and markedly narrower than palpomere 2, with a subapical seta on outer corner. Mentum nearly 1.5-times wider than long, parallel sided, moderately to strongly depressed anteromedially; anterior margin with relatively deep median excision, limited from the ventral surface by a U to V shaped transverse carina. Submentum rather flat; ocular ridge (see Komarek 2004, Fig. 1) well developed (see Fig. 4A). **Thorax.** Pronotum widest at base, narrowed anteriorly, surface rather evenly convex; ground punctation uniform, moderately fine, sometimes ground punctures connected by fine lines; seta bearing systematic punctures scattered through the surface, particularly noticeable as transverse anterolateral bands. Scutellum of moderate size, triangular, nearly as long as wide. Prosternum (Fig. 5A) well developed, flat, at most only weakly convex, not carinate; anterior margin of prosternum only slightly convex mesally; intercoxal process somewhat triangular (with base facing posteriorly), with surface posteriorly bifurcated. Mesoventrite not fused to mesepisterna, narrowly reaching anterior mesothoracic margin, posteriorly widely elevated; elevation usually with a transverse ridge, variable in shape and sharpness (in *Q. dentatus* the ridge is produced into a blunt, vertical, median tooth); mesepisternum obliquely widely concave. Mesofurca (examined in *Q. clusia* and *Q. reticulatus*; see Fig. 5B) with short arms, hardly as long as the length of mesocoxae; apex of arms triangular to irregularly explanate. Metaventrite weakly convex, medial posterior portion rather flat, entire metasternum very finely and densely pubescent, without median glabrous patch (reduced in *Q. acroreius* and *Q. dentatus*). Metepisterna approximately three times longer than wide, parallel-sided. Metafurca (examined in *Q. clusia* and *Q. reticulatus*; see Fig. 5C) short and stout, with furcal arms slightly longer than stalk; stalk somewhat triangular (wider near the crux, gradually narrowing distally); outer margins of stalk diverging from base towards midpoint of furcal arms; furcal arms somewhat rectangular, with apex (hemiductus) explanate, obliquely positioned; anterior tendons inserted near midpoint of dorsal edge of furcal arms; dorsal sheaths well developed, as wide as to slightly wider than widest point of lateral sheaths. **Elytra.** Surface even (without elevations or depressions), with 10 well defined longitudinal rows of serial punctures (see Fig. 6) (except in *Q. acroreius* and *Q. dentatus* which have irregularly punctate elytra; see Fig. 1), sutural series rather sharply impressed posteriorly, the remaining series slightly impressed; seta bearing systematic punctures scattered along interstriae; elytral margins slightly explanate anteriorly, increasing to more broadly explanate in posterior third. Epipleura well developed, densely covered by pubescence, rather weakly oblique, relatively wide anteriorly, gradually narrowing towards level of metacoxae, continued as a somewhat narrow stripe to apex; pseudepipleura glabrous, relatively wide throughout, only slightly narrowed posteriorly; surface of pseudepipleura smooth, undulated, anteriorly reticulate or posteriorly canaliculate. Wings (see Hansen 1999, fig. 17; Lawrence and Ślipiński 2013, fig. 23; examined in *Q. clusia* and *Q. reticulatus*) nearly three times longer than wide; radial cell as a pigmented, somewhat triangular area at anterior margin, positioned near mid length of wing; r4, RA_3+4_ and RA_3_ reduced; RA_3+4_ not connected to radial cell; RP_2_ reduced to a pigmented wide stipe; MP_3+4_, CuA_2_ and AA_3_ reaching margin of wing; basal cell long, reaching a little more than halfway towards posterior wing margin; wedge cell absent; anal (jugal) lobe well developed, narrow, demarcated from remainder of wing by a sharp excision at posterior wing margin. **Legs.** Pro- and mesofemora with dense pubescence, at most in about basal half, remainder of surface glabrous and shiny, with subtle reticulations; posterior femora mostly glabrous on ventral face, with only scarce scattered long setae over the surface, sometimes with reduced anterobasal, pubescent patch; all femora with rather sharp tibial grooves on inner face except basally. Tibiae moderately slender, rather weakly flattened, with moderately fine and sparse spines. All tarsi with five tarsomeres, bearing 2 (tarsomeres 2–4) to a few (tarsomere 5) long apical hair-like setae on dorsal face; tarsomere 5 without setae or spines on ventral face, tarsomeres 1–4 similar in size and shape; pro- and mesotarsi similar in size and proportions, tarsomeres 1–4 with moderately long and rather dense spiniform setae on ventral face, tarsomere 5 approximately as long as tarsomeres 1–4 combined; meta tarsi 1.3 times longer than pro- and mesotarsi, with tarsomeres 1–4 with 1–2 pairs of spines on ventral face, tarsomere 5 approximately as long as tarsomeres 2–4 combined; claws rather large, moderately curved. **Abdomen.** Abdomen with 5 ventrites, flat or very weakly convex, all ventrites with uniform, very fine and dense pubescence; first ventrite without median carina, posterior margin of fifth ventrite simply rounded. Aedeagus (Figs 7, 8) with basal piece about half the length of parameres; median lobe wider than base of each paramere, with a narrow, triangular, longitudinal sclerite, usually extending along apical third; parameres as long as, to longer than median lobe, and nearly half as wide; gonopore preapically situated; basal piece with lateral margins straight to sinuate, apically slightly diverging

**Larvae**: The immature stages are unknown.

##### Distribution.

**Figure 9.**
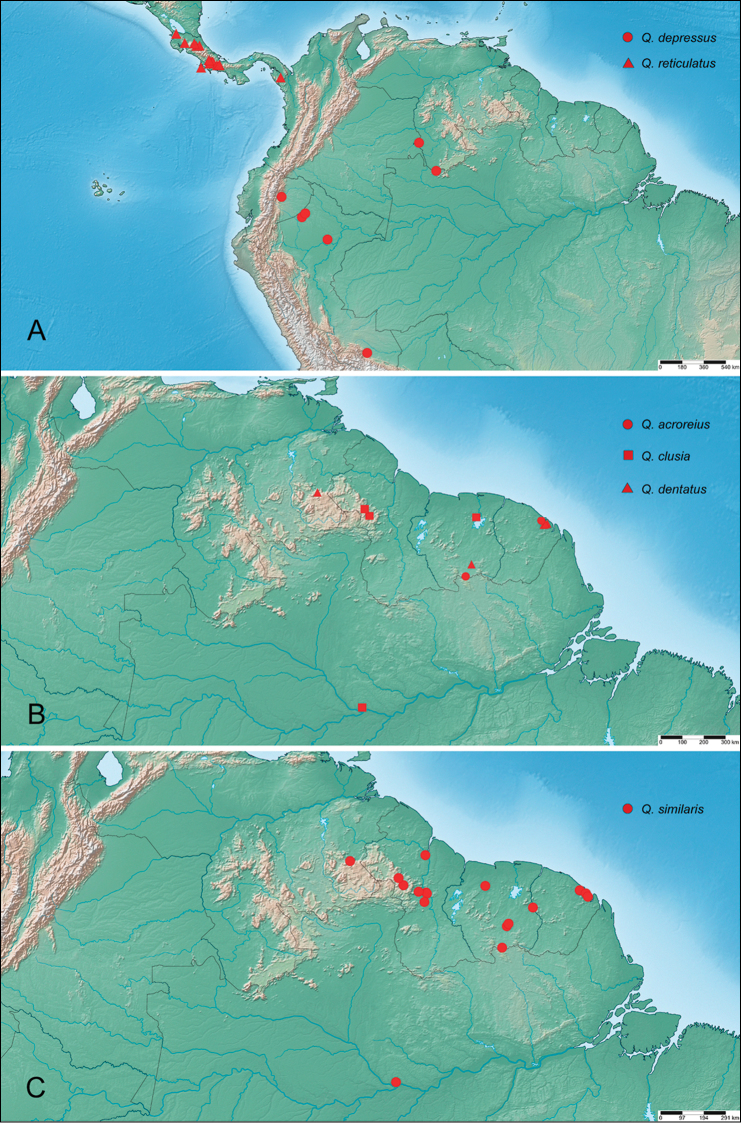
Distribution of *Quadriops* spp.: **A** *Q. depressus* (circles), *Q. reticulatus* (triangles) **B** *Q. acroreius* (circles), *Q. clusia* (squares), *Q. dentatus* (triangles) **C** *Q. similaris*.

Costa Rica (Cartago, Heredia, Limón, Puntarenas), Panama (Chiriquí, Darién), Ecuador (Napo), Peru (Loreto, Madre de Dios), Venezuela (Amazonas, Bolívar), Guyana, Suriname, French Guiana, Brazil (Amazonas). See Fig. 9B.

##### Biology.

Extensive collecting data as well as field observations confirm that the genus is terrestrial. While many specimens have been caught using flight intercept traps, many long series have been collected on decaying *Clusia* fruits. Additional specimens have been collected in rotten logs, sap flows on freshly cut trees, and in the refuse pile of leafcutter ants. The genus has never been collected from aquatic or semiaquatic habitats. It has been found at elevations from 30 to 1600 m. *Q. acroreius*, *Q. dentatus* and *Q. similaris* are not found higher than 350 m, whereas *Q. reticulatus* is usually found higher than 1000 m.

#### Quadriops acroreius sp. n.

Taxon classificationAnimaliaColeopteraHydrophilidae

http://zoobank.org/8E5BEE6A-5AC9-45DE-8C21-D3EB9BBE54B4

Figs 1E–H , 9B

##### Type material examined.

**Holotype (female).** “**SURINAME**: Sipaliwini District, 2°28'37.1994"N, 55°37'45.876"W, 275m/ Camp 1: Upper Palemeu,/ 10–16.iii.2012, leg. A.E.Z. Short/ Flight Intercept Trap/ SR12-0310-TN1” (SEMC; voucher SLE456). **Paratype (female)**: “**FRENCH GUIANA**, Cayenne, 33.5 km S and 8.4 km NW of Hwy N2 on Hwy D5, 30 m 4°48'18"N, 52°28'41"W, 29 MAY – 9 JUN 1997; J. Ashe, R. Brooks, FG1AB97 171 ex: flight intercept trap” // “Barcode/ SM0102412/ KUNHM-ENT” (SEMC, 1).

##### Differential diagnosis.

*Quadriops acroreius* is very similar to *Q. dentatus*, both species being moderately convex (as opposed to dorsally flattened) and the serial punctures of the elytra are randomly and uniformly distributed, not aligned to form well-defined longitudinal rows. It can be easily distinguished by the shape of the elevation of the mesoventrite, which is a wide, transverse, straight carina (as opposed to a toothlike projection as in *Q. dentatus*); in addition, the surface of head and clypeus between punctures is smooth (as opposed to reticulated).

##### Description.

Body length 1.9–2.0 mm, width 1.2–1.3 mm. Body elongate oval, moderately convex. General coloration uniform dark brown. Surface of pronotum and elytra, smooth (as opposed to reticulated between punctures), only slightly reticulated on head and clypeus. Elevation of mesoventrite forming a wide, transverse, straight, blunt, strongly raised carina. Metaventrite with a postero median semi triangular glabrous area. Elytra with randomly and uniformly distributed punctures, not aligned into striae; surface of pseudepipleura anteriorly reticulated, posteriorly smooth. Metafemora with basal 1/8 covered by pubescence.

##### Etymology.

Named from the Greek “*akroreia*”, meaning mountain ridge (Brown 1956), in reference to the pronounced transverse carina on the elevation of the mesoventrite.

##### Distribution.

Suriname; French Guiana. See Fig. 9B.

##### Biology.

The male of the species is not known. Specimens were collected at flight intercept traps.

#### Quadriops clusia sp. n.

Taxon classificationAnimaliaColeopteraHydrophilidae

http://zoobank.org/D68D2596-CAE0-4BDE-A46C-F84CA400D8F4

Figs 3E–H , 6A , 7I–K , 9B

##### Material examined.

**Holotype (male).** “SURINAME: Brokopondo District/ 4.95069'N, -55.18599, 470 m/ Brownsberg Nature Park, Leo Val trail, nr. Pump station/ rotting Clusia fruits; 22.iii.2017/ leg. Short et al., SR17-0322-03A // Barcode: SEMC1542023” (NZCS). **Paratypes (210 exs.): BRAZIL: Amazonas**: Reserva Ducke 26 km NE Manaus, Barbosa, M.G.V., Plot B, FIT 1, Feb 1995 (1 male, dissected, NHMUK). **GUYANA: Region XIII**: 5°0.673'N, 59°38.358'W, 500 m, Upper Potaro Camp I (c. 7 km NW Chenapau), near camp, rotten fruits of *Clusia*; leg. A. Short, 12.iii.2014, GY14-0312-04A // Barcodes: SEMC1315733–37, 39, 42–46, 48, 52–55, 57, 60–61, 65, 67–69 (23 ex., incl. 1 female, 7 males [SEMC1315754 dissected]), SEMC1328917–45, 49–60, 62–65, 67-76, 78–85 (SEMC, CBDG, 63 ex., incl. 5 females [SEMC1328965, 78 dissected], 14 males [SEMC1328983 dissected], SEMC1329066–72, 74–75 (SEMC, 9, incl 2 males); 5°18.261'N, 59°50.257'W, 687 m, Ayanganna Airstrip, trail from airstrip to Ayanganna, rotten fruits of *Clusia*; leg. A. Short, 17.iii.2014, GY14-0317-01B // Barcodes: SEMC1329083–84, 86–87, 89–90, 92–93, 96–97 (10, incl. 2 females SEMC1329090 dissected], 5 males [SEMC1329093 dissected], voucher SLE 1003, voucher SLE 1077, voucher SLE 1078; **SURINAME: Brokopondo**: 4.95069'N, -55.18599, 470 m/ Brownsberg Nature Park, Leo Val trail, nr. Pump station/ rotting Clusia fruits; 22.iii.2017, leg. Short et al., SR17-0322-03A // Barcodes: SEMC1541993–2022; 2024 – 2119 (126 ex., SEMC, NZCS, MIZA). **Sipaliwini**: Raleighvallen Nature Reserve/ Lolopasie Area, 14.iii.2016, leg Short et al., *Clusia* fruits, SR16-0314-02B (SEMC; voucher SLE 1054); 4°42'28.8", -56°13'9.5448"; 24 m/ Raleighvallen Nature Reserve/ Lolopasie Area; 18.iii.2016/ *Clusia* fruits; leg. Short/ SR16-0318-01C (SEMC; voucher SLE 1071, female).

##### Differential diagnosis.

*Quadriops clusia* has well defined longitudinal rows of serial punctures (as opposed to uniform and randomly distributed as in *Q. acroreius* and *Q. dentatus*, see Fig. 1). The serial punctures on the striae are simple and similar in size as those on the interstria (Fig. 6A) (as opposed to ramified and conspicuously larger than the punctures on the interstrial surface, as on the remainder species, see Fig. 6B). Transverse ridge on the elevation of the mesoventrite rather blunt, and slightly bisinuate.

##### Description.

Body length 2.1–2.5 mm, width 1.2–1.4 mm. Body elongate oval, moderately and evenly convex. General coloration reddish brown, with pronotum and clypeus only slightly paler. Surface of head, frons and pronotum reticulated. Clypeus with anterior margin nearly straight. Elevation of mesoventrite with transverse ridge rather broad, and slightly bisinuate. Elytra with ten well defined longitudinal rows of serial punctures; punctures on the interstrial surface similar in size to serial punctures (Fig. 6A); surface of pseudepipleura anteriorly undulated, particularly at limit with epipleura, posteriorly smooth. Metafemora with pubescence only along articulation with trochanter, and sometimes along proximal 1/6 of anterior margin. Aedeagus (Figs 7I–K) with parameres as long as median lobe, and nearly as wide at apical 1/4; parameres with outer margins nearly straight, only slightly curved inwards at apical 1/3; apical 1/3 of inner margin of parameres concave; apical 1/3 of parameres rather digitiform and straight, parallel to longitudinal axis of aedeagus. Median lobe with lateral margins straight, converging towards the apex; apex of aedeagus widely rounded; gonopore rather semicircular. Basal piece as long as 0.5-times the length of the median lobe, with lateral margins straight; manubrium 0.5 times the length and nearly as wide as the base of basal piece.

##### Etymology.

Named after *Clusia*, the genus of plants on whose decomposing fruits the beetles have been collected.

##### Variation.

There is slight variation in the proportions of the aedeagus. Some specimens might have a comparatively wider median lobe (Fig. 7K), or seem more slender overall (Fig. 7J).

##### Distribution.

Brazil (Amazonas), Guyana, Suriname. See Fig. 9B.

##### Biology.

**Figure 10.**
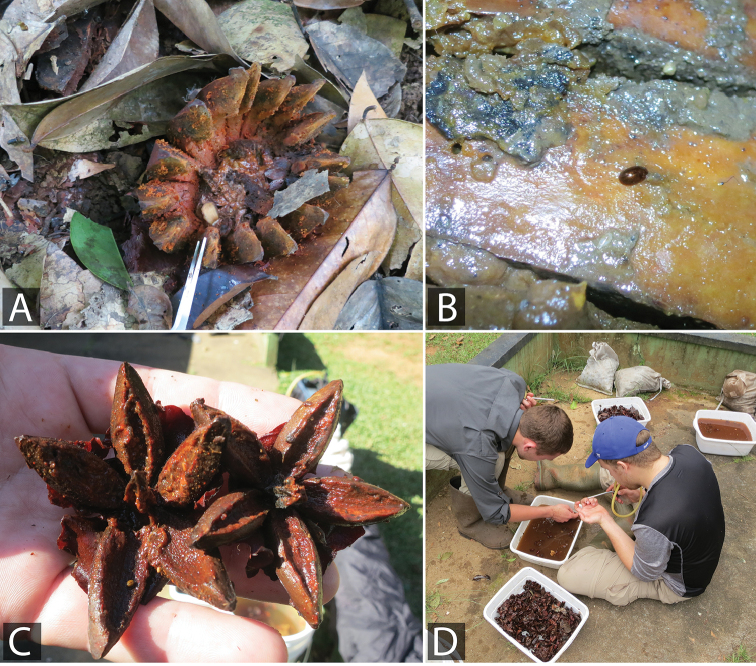
Habitat of *Quadriops clusia* sp. n.: **A** Clusia cf. grandiflora on the forest floor, collecting event GY14-0312-04A **B** A specimen of *Quadriops clusia* sp. n. crawling on the surface of a rotting *Clusia* fruit, collecting event GY14-0312-04A **C** Clusia cf. nemorosa in Brownsberg Nature Park, Suriname on which *Quadriops clusia* sp. n. was collected **D** Collecting *Quadriops* and other terrestrial hydrophilid specimens by submerging collected rotting *Clusia* fruits in pans of water and waiting for the beetles to float to the surface, collecting event SR17-0322-03A.

Most known specimens have been collected on rotten fruits of *Clusia* trees, sometimes in series of many hundreds of individuals. In Guyana, this species was found on and beneath rotten fruits of *Clusia grandiflora* (Fig. 10A–B). In Suriname, this species was collected on and beneath the rotten fruits of several *Clusia* species, including *C. grandiflora* and C. cf. nemorosa (Fig. 10C–D). The beetles appear most common on fruits in a stage of decay where they are soft and sticky (as opposed to more advanced stages of decay in which the fruits become dry or crumbly). The beetles were also present in leaves beneath the decaying fruits into which rotting fluids had seeped. Most specimens were collected by collecting these fruits and submerging them in pans of water, at which time the beetles float to the surface. We collected hundreds of specimens on several occasions using this method. However, not all rotten *Clusia* patches we examined (some even within 1 km of other patches with *Quadriops* abundance) contained many or any *Quadriops* specimens. We sifted general forest litter and did extensive aquatic collecting at sites in Guyana and Suriname where we found abundant *Quadriops clusia* populations, but no specimens were ever found in these habitats. We also laid baits of other fruits including bush cashews and bananas but these were not successful in attracting *Quadriops*. We believe the habitat of this species is likely restricted to rotten fruits, and possibly only those from *Clusia*. *Quadriops clusia* has been collected at elevations between 500 and 700 m.

#### Quadriops dentatus

Taxon classificationAnimaliaColeopteraHydrophilidae

Hansen, 1999

Figs 1A–D , 9B

Quadriops dentatus Hansen, 1999: 134.

##### Type material examined.

**Holotype (female)**: “VENEZ [Venezuela] : Bolivar/ 105 km S El Dorado/ 17.VII–7.VIII.86/ B. Gill 350m”, “Flight intercept/ trap”, “HOLOTYPE”, “[Handwritten] HOLOTYPE/ Quadriops dentatus/ M. Hansen”, “[Barcode] Canadian Museum of/ Musée canadien de la/ NATURE/ CMNEN 0011502” (CMNC).

##### Additional material examined

**(5 exs.). FRENCH GUIANA: Matoury**: 41.5 km SSW on Hwy N2, 4°37'22"N, 52°22'35W, 50m, 29 May–9 Jun 1997, J. Ashe, R. Brooks, FG1A97 170, ex: flight intercept trap // Barcodes: SM0134289 (SEMC, 1 female), SM0134241 (SEMC, 1 female, dissected); **Roura**: 8.4 km SSE, 200 m, 4°40'41"N, 52°13'25"W, 25–29 May 1997, J. Ashe, R. Brooks, FG1AB97 088, ex: flight intercept trap // Barcode: SM0096111 (SEMC, 1 female); 13.0 km SSE, 240 m, 4°38'38"N, 52°17'56"W/ 13 Jun 1997; J. Ashe, R. Brooks, FG1AB97 196, ex: miscellaneous collecting // Barcode: SM0100061 (SEMC, 1 female). **SURINAME: Sipaliwini**: Camp 4 (low), Kasikasima, 2.97731°N, 55.38500°W, 200m, 20–25 mar 2012, leg. Larsen, flight intercept trap, SR12-0320-TN1, 2012 CI-RAP Survey // Barcode: SEMC1089659 (SEMC, 1 female).

##### Differential diagnosis.

*Quadriops dentatus* is very similar to *Q. acroreius*, both species being moderately convex (as opposed to dorsally flattened) and the serial punctures of the elytra are randomly and uniformly distributed, not aligned to form well defined longitudinal rows. It can be easily distinguished by the toothlike projection of the mesoventrite (as opposed to a wide, transverse, straight, blunt carina as in *Q. acroreius*); in addition, the surface of head and clypeus is smooth between punctures (as opposed to reticulated).

##### Redescription.

Body length 1.6–2.2 mm, width 1.1–1.2 mm. Body elongate oval, moderately convex. General coloration uniform yellowish to dark brown. Surface smooth (as opposed to reticulated between punctures) on head, pronotum and elytra. Elevation of mesoventrite forming a basally transverse acute tooth. Metaventrite with a posterior, short, glabrous and narrow stripe. Elytra with randomly and uniformly distributed punctures, not aligned into striae; surface of pseudepipleura smooth throughout, at most only slightly reticulated at base. Metafemora with pubescence only along dorsal area of articulation to trochanter.

##### Variation.

There is variation in size with the type specimen being the largest.

##### Distribution.

Venezuela (Bolívar), Suriname (Sipaliwini), French Guiana (Matoury, Roura). See Fig. 9B.

##### Biology.

The male of this species remains unknown. All known specimens were collected using flight intercept traps, at elevations between 50 and 350 m.

#### Quadriops depressus

Taxon classificationAnimaliaColeopteraHydrophilidae

Hansen, 1999

Figs 2A–D , 7A–D , 9A

Quadriops amazonensis García, 2000: 59, **syn. n.**
Quadriops depressus Hansen, 1999: 136.
Quadriops politus Hansen, 1999: 135, **syn. n.**

##### Type material examined.

**Holotype (male): *Q. depressus***: “PERU: Dept. [Departamento] Loreto/ 1.5km N Teniente Lopez/ 2°35.66'S,76°06.92'W/ 22 July 1993, 210–240 m/ Richard Leschen #164/ ex: flight intercept trap”, “[Handwritten] PARATYPE/ Quadriops depressus/ M. Hansen”, “[Barcode]/ SEMC0965921/ KUNHM-ENT” (SEMC). **Paratypes: *Q. depressus***: “PERU: Dept. [Departamento] Loreto/ Teniente Lopez/ 2°35.66'S,76°06.94'W/ 23 July 1993, 210–240 m/ Richard Leschen #191/ ex flight intercept trap”, “[Handwritten] HOLOTYPE/ Quadriops depressus/ M. Hansen” (SEMC); “PERU: Dept. [Departamento] Loreto/ Campamento San Jacinto/ 2°18.75'S, 75°51.77'W/ 11 July 1993, 175–215 m/ Richard Leschen #84/ ex: flight intercept trap”, “[Handwritten] PARATYPE/ Quadriops depressus/ M. Hansen” (SEMC).

***Q. amazonensis*: Holotype (male)**: “Venezuela, Amazonas,/ Mcipio. [Municipio] Guinia, Yavita,/ Caño Chivichi, 600 m,/ 29–31 / VIII/ 1996 / Trampa interceptación”, “Colector:/ J. Camacho”, “Holotipo [male symbol, handwritten]/ [Handwritten] Quadriops/ [Handwritten] amazonensis/ Dcrip. M. García, 1998”, “[Barcode]/ MALUZ10158/ LUZ-Venezuela”. (MALUZ).

***Q. politus*: Holotype (male)**: “PERU: Dept. [Departamento] Loreto/ Campamento San Jacinto/ 2°18.75'S, 75°51.77'W/ 11 July 1993, 175–215 m/ Richard Leschen #83/ ex: flight intercept trap”, “[Handwritten] HOLOTYPE/ Quadriops politus/ M. Hansen”, “[Barcode]/ SEMC0965917/ KUNHM-ENT” (SEMC). **Paratype**: “PERU: Dept. [Departamento] Loreto/ Campamento San Jacinto/ 2°18.75'S, 75°51.77'W/ 11 July 1993, 175–215 m/ Richard Leschen #83/ ex: flight intercept trap”, “[Handwritten] PARATYPE/ Quadriops politus/ M. Hansen”, “[Barcode]/ SEMC0965917/ KUNHM-ENT” (SEMC).

##### Additional material examined

**(3 exs.). PERU: Loreto**: Campamento San Jacinto, 2°18.75'S, 75°51.77'W, 11 July 1993, 175–215 m/ Richard Leschen #82/ ex: flight intercept trap (SEMC, 1); **Madre de Dios**: Pantiacolla Lodge, 8 km NW El Mirador Trail, Alto Madre de Dios River, 800 m, 12°38'30"S, 71°16'41"W, 23–26 OCT 2000, R. Brooks, PERU1B00 102, ex. flight intercept trap // “Barcode/ SM0260334/ KUNHM-ENT” (SEMC, 1 female). **VENEZUELA: Amazonas**: T. F. Amaz. Cerro de la Neblina Basecamp, 140 m, 0°50'N, 66°10'W, 10–20 February 1985// Flight intercept pan trap in rainforest, P. J. & P. M. Spangler, R. A. Faitoute, W. E. Steiner colrs.” (1 male, USNM).

##### Differential diagnosis.

*Quadriops depressus* is externally very similar to *Q. similaris*, and *Q. reticulatus*, as all have well defined longitudinal rows of serial punctures (as opposed to uniform and randomly distributed as in *Q. acroreius* and *Q. dentatus*, see Fig. 1), and the serial punctures are conspicuously larger than the punctures on the interstrial surface (as opposed to similarly large as in *Q. clusia*, see Fig. 6). It can be separated from *Q. reticulatus* by the dorsal outline of the body being nearly flat (as opposed to moderately convex), and the surface of the pseudepipleura posteriorly markedly canaliculated (see Fig. 2C; as opposed to smooth). *Quadriops depressus* can be distinguished from *Q. similaris* by the rounded shape of the apex of the parameres (see Fig. 7 A–D; as opposed to angulate, as in Fig. 8).

##### Redescription.

Body length 2.1–2.5 mm, width 1.3–1.4 mm. Body elongate oval, moderately convex, with dorsal outline nearly flat. General coloration reddish to dark brown, with pronotum and clypeus only slightly paler. Surface of clypeus smooth to reticulated, usually smooth on frons and pronotum. Elevation of mesoventrite with transverse ridge rather fine and curved (posteriorly concave). Elytra with ten well defined longitudinal rows of serial punctures; punctures on the interstrial surface noticeably smaller than serial punctures; surface of pseudepipleura anteriorly undulated, particularly at limit with epipleura, posteriorly reticulated. Metafemora with pubescence only at base of anterior margin at most. Aedeagus (Figs 7A–D) with parameres slightly longer than median lobe; parameres with outer margins nearly straight, only slightly curved inwards at apical 1/3; apical 1/3 of inner margin of parameres concave; apical 1/6 of parameres digitiform, with rounded apex slightly directed towards longitudinal axis of aedeagus. Median lobe with lateral margins straight, slightly converging from base; apex of aedeagus widely rounded; gonopore rather semicircular. Basal piece as long as 0.4-times the length of the median lobe, with lateral margins straight; manubrium 0.5-times the length and clearly narrower than the base of basal piece.

##### Variation.

There is variation on the density and presence of reticulation on the surface of the frons and clypeus.

##### Distribution.

Peru (Loreto), Ecuador (Napo), Venezuela (Amazonas). See Fig. 9B.

##### Biology.

All known specimens were collected with flight intercept traps.

##### Remarks.

It is known that the shape of the aedeagus exhibit intraspecific variation in several acidocerine species (see Short et al. 2017), including *Q. similaris* below. Given the similarity of the illustrations provided by Hansen (1999) and García (2000), and the lack of external characters (beyond the density and presence of reticulation on the surface of the frons and clypeus) to clearly distinguish *Q. amazonensis* and *Q. politus* from *Q. depressus*, we dismounted the aedeagi of the holotypes of the latter two species to compare them directly. The aedeaegus of all three type specimens, along with an additional male from Venezuela, share the same overall shape, with only slight variations in proportions that may be due in part to the preparation of the structures. Further, we found the illustration of the aedeagus of the type of *Q. politus* to be incorrectly interpreted (Hansen 1999: fig. 24); specifically, the apex of the median lobe is drawn to appear very constricted and acutely pointed, while in fact it is broad and blunt as with most other *Quadriops*. This apex of the median lobe was strongly cleared in the type and was not readily visible until we dismounted the genitalia to view it with transmitted light. This mistaken interpretation of the aedeagal shape seems likely to be why Hansen had considered this to be a separate species when in fact it does not appear so. Given the similarity of the aedeagi, coupled with the lack of any detectable external differences, we consider *Q. amazonensis* and *Q. politus* both junior synonyms of *Q. depressus*.

#### Quadriops reticulatus

Taxon classificationAnimaliaColeopteraHydrophilidae

Hansen, 1999

Figs 3A–D , 5 , 6B , 7E–H , 9A

Quadriops reticulatus Hansen, 1999: 135

##### Type material examined.

**Holotype (male)**: “COSTA RICA: Puntarenas/ Las Alturas (Stanford/ Biol. [Biological] Sta. [Station]) ca. 29km NE San/ Vito, 1500m, 27 May 1993/ J.S. & A.K. Ashe #063/ ex: flight intercept trap”, “[Handwritten] HOLOTYPE/ Quadriops reticulatus/ M. Hansen”, “[Barcode]/ SEMC0965918/ KUNHM-ENT” (SEMC).

##### Additional material examined

**(135 exs.). COSTA RICA: Cartago**: 19.3 km NE San José, 17 May 1993, 1010 m, J. & A. Ashe, #105, ex: flight intercept trap (SEMC, 1); Tapanti National Park, 9.776711, -83.792778, Kiri Lodge, 14-19.vii.2011, leg. Short et al., Flight intercept trap, CR11-FIT-Z1-A1 (SEMC; voucher SLE 401). **Guanacaste**: Est. Pitilla, 9 km S. Sta. Cecilia, 700 m, P. N. Guanacaste, P. Ríos, Set. 1991 (INBIO, 1 male); same, 9–20 Nov 1993, C. Moraga, L N 330200_380200 #2449 (INBIO, 1); **Heredia**: 16 km SSE La Virgen, 1070 m, 10°16'N, 84°05'W, 11–20.ii.2001, 11/TN/08/003/ INBio-OET-ALAS transect// Barcodes: SEMC0859232–33 (SEMC, 2 males); same, 11/TN/09/004 // Barcodes: SEMC0859174, 184–185, 203, 206–207 (SEMC, 6 exs., incl. 3 females, 1 male); same, 11/TN/16/006 // Barcodes: SEMC0860054, 58, 68, 73–74, 76 (SEMC, 6 exs., incl. 4 females, 2 males); same, 11/TN/17/007 // Barcodes: SEMC0859642, 827, 836, 840 (SEMC, 4 exs., incl. 2 females, 1 male); same, 11/TN/20/010 // Barcodes: SEMC0860259, 272 (SEMC, 2 exs., incl. 1 female, 1 male); same, 10–21.iii.2001, 11/TN/06/011, // Barcodes: SEMC0859457, 469, 496, 498, 536 (SEMC, 5 males); same, 11/TN/07/012 // Barcodes: SEMC0859353, 363, 374, 385, 389, 599 (6 exs., incl. 2 females, 4 males); same, 11/TN/10/015 // Barcodes: SEMC0859524, 551 (SEMC, 2 exs., incl. 1 female [dissected], 1 male); same, 11/TN/16/016 // Barcodes: SEMC0859973, 975, 979, 0860000, 010, 012 (SEMC, 6 exs., incl. 4 females [SEMC0860000 dissected], 2 males [SEMC0859979 dissected]; same, 11/TN/18/018 // Barcodes: SEMC0859708, 712, 718, 721, 728–729, 733, 738–739, 750, 756, 765, 776, 783, 795 (SEMC, 15 exs., incl. 6 females [SEMC0859712 dissected], 6 males); same, 11/TN/19/019 // Barcodes: SEMC0860511, 515, 527, 531 (SEMC, 4 exs., (2 females, 2 males); same, 11/TN/20/020 // Barcode: SEMC0859935 (SEMC, 1 female); same, 10–21.iv.2001, 11/TN/07/022 // Barcodes: SEMC0860215–216, 220, 226, 233, 254 (SEMC, 6 exs., incl. 4 females, 1 male); 11/TN/08/023 // Barcodes: SEMC0859257, 264, 268–269 (SEMC, 4 exs., incl. 1 female, 2 males [SEMC0859268 and SEMC0859269 dissected]; same, 11/TN/16/026 // Barcodes: SEMC0859406–408, 419, 431, 437 (SEMC, 6 exs., 5 females, 1 dissected male [SEMC0859437]; same, 11/TN/17/027 // Barcodes: SEMC0860628, 630, 641, 652, 663 (SEMC, 5 exs., 1 female, 4 males); same, 11/TN/18/028 // Barcodes: SEMC0860126, 137, 146, 148, 152 (5 exs., 2 females, 3 males); same, 11/TN/19/029 // Barcodes: SEMC0859270, 273, 284, 306, 308, 314, 325 (SEMC, 7 exs., incl. 4 females, 2 males); same, 11/TN/20/030 // Barcodes: SEMC0860573, 599, 602, 606–607, 620, 624–625, 0892500, 502, 511, 578, 583, 590–591, 594 (SEMC, 16 exs., incl. 5 females [SEMC0860599, SEMC0860606 and SEMC0860620 dissected] 5 males [SEMC0860573 and SEMC0860607 dissected]; same, 11–20.iv.2001, 11/TN/18/008 // Barcodes: SEMC0859660, 663–664, 666, 675, 677, 683–684, 704 (SEMC, 9 exs., incl. 4 females, 1 male); same, 12–23.iii.2003, 05/TN/18/020 // Barcode: SEMC0860494 (1 female); same, 10–22.iii.2004, 03/TN/08/015 // Barcode: SEMC0860372 (SEMC, 1); **Limón**: Sector Cerro Cocori, Fca. de E. Rojas, 150 m, E. Rojas, Oct 1991 (INBIO, 1 male); **Puntarenas**: Monteverde Biological Preserve Peñas Blancas Valley- Aleman refugio, 25–29 May 1993, Steve Lingafelter, ex: flight intercept trap (SEMC, 1); Corcovado National Park, Sirena Stn., Corcovado Trail, 150 m, 8°29'7"N 83°34'39"W, 28 JUN–1 JUL 2000, Z. H. Falin, CR1ABF00 059, ex: flight intercept trap // Barcodes: SM0251795 (SEMC, 1 male, dissected), SM0251874 (SEMC, 1 female, dissected), SM0252279 (SEMC, 1 female); Las Cruces Biol. Sta., 1330 m, 08°47.14'N 82°57.58'W, 28–30-V-2004, J. S. Ashe, Z. Falin, I. Hinojosa, ex: flight intercept trap, CR1AFH04 059 // Barcode: SM0625787 (SEMC, 1 male); Altamira Biol. Sta., 1510–1600 m, 09°01.76'N 83°00.49'W, 4–7-VI-2004, J. S. Ashe, Z. Falin, I. Hinojosa, ex: flight intercept trap, CR1AFH04 144 // Barcode: SM0659727 (SEMC, 1). **PANAMA: Chiriqui**: 20 Km N Gualaca, Finca La Suiza, 1350 m, 08°39'N, 82°12'W, 10 June 1995, J. Ashe & R. Brooks, #167, ex: fogging fungusy log (SEMC, 2 [1 male, dissected]); La Fortuna, “Hydro. Trail”, 08°42'N, 82°14'W, 1150 m, 23 V–9 VI 1995, J. Ashe, R. Brooks, #156, ex: flight intercept trap (SEMC, 2 [1 female]); **Darién**: Cana Biological Station, 550 m 7°45'18"N, 77°41'6"W, 07–09 Jun 1996, J. Ashe, R. Brooks, PAN1AB96 114, ex: flight intercept trap // Barcode: SM0049123 (SEMC, 1 female).

##### Differential Diagnosis.

*Quadriops reticulatus* is externally very similar to *Q. depressus*, and *Q. similaris*, as all have well defined longitudinal rows of serial punctures (as opposed to uniform and randomly distributed as in *Q. acroreius* and *Q. dentatus*), and the serial punctures are conspicuously larger than the punctures on the interstrial surface (as opposed to similarly large as in *Q. clusia*, see Fig. 6). It can be separated from *Q. depressus* and *Q. similaris* by the dorsal outline of the body being uniformly convex (as opposed to flat), and the smooth surface of the pseudepipleura (as opposed to posteriorly markedly canaliculated, see Fig. 2C and G).

##### Redescription.

Body length 2.0–2.4 mm, width 1.2–1.45 mm. Body elongate oval, moderately convex, with dorsal outline only slightly flat. General coloration reddish to dark brown, with margins of pronotum and clypeus only slightly paler. Surface of clypeus, frons and pronotum reticulated. Elevation of mesoventrite with transverse ridge rather fine and curved (posteriorly concave). Elytra with ten well defined longitudinal rows of serial punctures; punctures on the interstrial surface noticeably smaller than serial punctures; surface of pseudepipleura anteriorly undulated, particularly at limit with epipleura, posteriorly smooth. Metafemora with pubescence only on anterior basal corner. Aedeagus (Fig. 7E–H) with parameres as long as or longer than median lobe; parameres with outer margins slightly concave near midlength; apical 1/3 of inner margin of parameres concave; apical 1/3 of parameres digitiform, rather narrow, with rounded apex, slightly pointing towards longitudinal axis of aedeagus. Median lobe with lateral margins usually straight and clearly converging from base; apex of aedeagus widely rounded; gonopore variable in shape. Basal piece as long as 0.5 to 0.7-times the length of the median lobe, with lateral margins straight to sinuate; manubrium 0.3 to 0.6-times the length and clearly narrower than the basal piece at its base.

##### Variation.

The degree of sharpness of the transverse ridge of mesoventrite varies from being blunt and moderately marked to sharp. There is variation on the shape of the aedeagus, even though the overall shape is conserved across the species. Specimens from Panama tend to be smaller.

##### Distribution.

Costa Rica: Cartago, Heredia, Limón, Puntarenas; Panama: Chiriquí, Darién. See Fig. 9B.

##### Biology.

Most known specimens have been collected by using flight intercept traps. A few specimens were collected from “fungusy logs”. Additionally, a disassociated note at INBio about one collecting event of *Quadriops* in Costa Rica indicated a series had been collected on the sap of freshly cut trees. Most *Q. reticulatus* specimens have been collected at elevations between 1000 and 1600 m.

##### Remarks.

The female specimen from the Darién of Panama has a differently shaped transversal ridge of the mesoventrite, but no other characters were found to differentiate it from *Q. reticulatus*. However, when males are found it may be shown to represent a distinct species. Several specimens were also observed to have mites on the dorsal surface of the elytra.

#### Quadriops similaris

Taxon classificationAnimaliaColeopteraHydrophilidae

Hansen, 1999

Figs 2E–H , 8 , 9C

Quadriops similaris Hansen, 1999: 136

##### Type material examined.

**Paratype (female)**: “VENEZ [Venezuela]: Bolivar/ 105 km S El Dorado/ 17.VII-7.VIII.86/ B. Gill, FIT [handwritten] 350m”, “Flight intercept/ trap”, “PARATYPE”, “[Handwritten] PARATYPE/ Quadriops/ similaris/ M. Hansen”, “CMNEN 2003-1173” (CMNC). [Holotype female from Guyana in NHMUK, not examined].

##### Additional material examined

**(34 exs.). BRAZIL: Amazonas**: Reserva Ducke 26 km NE Manaus, Barbosa, M.G.V., Plot B, FIT 1, Feb 1995 (2 males (1 dissected); 1 female, NHMUK). **GUYANA: Region 8**: Iwokrama Forest, Pakatau hills, 70 m, 4°44'54"N, 59°1'36"W, 25–29 MAY 2001, R. Brooks, Z. Falin, GUY1BF01 061, ex: flight intercept trap // Barcode: SM0569493 (SEMC, 1); Iwokrama Forest, 1 km W Kurupukari, Iwokrama Field Stn., 60 m, 4°40'19"N, 58°41'4"W, 21 MAY 2001, R. Brooks, Z. Falin, GUY1BF01 005, ex: *Acromyrmex hystrix* refuse pile // Barcodes: SM0569493 (SEMC, 1); SM0568525 (SEMC, 1 male, dissected); SM0568547 (SEMC, 1 female, dissected); 26–29 MAY 2001, R. Brooks, Z. Falin, GUY1BF01 064, ex: flight intercept trap // Barcode: SM0569493 (SEMC, 1); Iwokrama Forest, 26 km SW Kurupukari, Iwokrama Mt., 300 m, 4°20'2"N, 58°47'18"W, 23–25 MAY 2001, R. Brooks, Z. Falin, GUY1BF01 031, ex: flight intercept trap // Barcode: SM0570965 (SEMC, 1); Iwokrama Forest, Turtle Mt. base camp, 50 m, 4°43'5"N, 58°43'5"W, 1 JUN 2001, R. Brooks, Z. Falin, GUY1BF01 105, ex: fogging splintered tree trunk // Barcodes: SM0564705 (SEMC, 1 male, dissected), SM0564703 (SEMC, 1 male), SM0564690 (SEMC, 1 male), SM0564710 (SEMC, 1), SM0564711 (SEMC, 1), SM0564721 (SEMC, 1). **FRENCH GUIANA: Cayenne**: 33.5 km S and 8.4 km NW of Hwy N2 on Hwy D5, 4°48'18"N, 52°28'41"W, 30 m, 26–28 MAY 1997; J. Ashe, R. Brooks, FG1AB97 057, ex: flight intercept trap // Barcode: SM0099106 (SEMC, 1 female, dissected); 29 MAY–9 JUN 1997; J. Ashe, R. Brooks, FG1AB97 171, ex: flight intercept trap // Barcode: SM0102330 (SEMC, 1 male, dissected), SM0101058 (SEMC, 1 female), SM0131132 (SEMC, 1 male, dissected); **Roura**: 8.4 km SSE, 200 m, 4°40'41"N, 52°13'25"W, 22–24 MAY 1997; J. Ashe, R. Brooks, FG1AB97 027, ex: flight intercept trap // Barcodes: SM0101145 (SEMC, 1 female, dissected); SM0101159 (SEMC, 1 male, dissected); 4°40'0"N, 52°13'0"W, 29 MAY–10 JUN 1997; J. Ashe, R. Brooks, FG1AB97 182, ex: flight intercept trap // Barcodes: SM0121078 (SEMC, 1); SM0121118 (SEMC, 1 female); 39.4 km SSE, 270 m, 4°32'43"N, 52°8'26"W, 10 JUN 1997; J. Ashe, R. Brooks, FG1AB97 173, ex: under fermenting bark // Barcode: SM0098799 (SEMC, 1 female). **SURINAME: Saramacca**: West Suriname Road, 178 km WSW Zanderij Airport, 25 m, 4°59'6"N, 56°18'48"W, 13 JUN 1999; Z. H. Falin; SUR1F99 070, ex: splintered tree trunk (pyrethrum fogging) // Barcodes: SM0181529 (SEMC, 1), SM0181531 (SEMC, 1 female, dissected); SM0181968 (SEMC, 1 male, dissected); **Sipaliwini**: Palumeu, 15 km NE, on Tapanahony River, trail to Poti Hill, ca 160 m, 3°27'N, 55°22'W, 6 JUL 1999; Z. H. Falin; SUR1F99 164, ex: splintered tree trunk (pyrethrum fogging) // Barcode: SM0184065 (SEMC, 1 male); Palumeu, ca 160 m, 3°20'56"N, 55°26'18"W, 8 JUL 1999; Z. H. Falin; SUR1F99 182, ex: splintered log (pyrethrum fogging) // Barcodes: SM0180696 (SEMC, 1), SM0180698 (SEMC, 1), SM0180699 (SEMC, 1 female, dissected); N 2.47700°, W 55.62941°, 275 m, Camp 1, Upper Palumeu, leg. A. Short; Flight Intercept Trap, 10–16.iii.2012; SR12-0310-TN1, 2012 CI-RAP Survey // Barcode: SEMC1089282 (SEMC, 1).

##### Differential diagnosis.

*Quadriops similaris* is externally very similar to *Q. depressus* and *Q. reticulatus*, as all have well defined longitudinal rows of serial punctures (as opposed to uniform and randomly distributed as in *Q. acroreius* and *Q. dentatus*, see Fig. 1), and the serial punctures are conspicuously larger than the punctures on the interstrial surface (as opposed to similarly large as in *Q. clusia*, see Fig. 6). It can be separated from *Q. reticulatus* by the dorsal outline of the body being nearly flat (as opposed to uniformly convex), and the surface of the pseudepipleura posteriorly markedly canaliculated (see Fig. 2G; as opposed to smooth). *Q. similaris* can be distinguished from *Q. depressus* by the angulate shape of the apex of the parameres (see Fig. 8; as opposed to rounded, as in Fig. 7A–D).

##### Redescription.

Body length 2.2–2.6 mm, width 1.4–1.6 mm. Body elongate oval, moderately convex, with dorsal outline nearly flat. General coloration reddish brown, with sides of pronotum and clypeus only slightly paler. Surface of clypeus, frons and sides of pronotum reticulated. Elevation of mesoventrite with transverse ridge usually rather sharp, curved to somewhat roof-like shaped. Elytra with ten well defined longitudinal rows of serial punctures; punctures on the interstrial surface noticeably smaller than serial punctures; surface of pseudepipleura anteriorly undulated, posteriorly markedly canaliculated. Metafemora with pubescence only along proximal 1/5 of anterior margin. Aedeagus (Fig. 8) with parameres usually longer than median lobe; parameres with outer margins straight to slightly convex along basal 3/4, then distinctly curved inwards at apical 1/4, or gradually curved from midlength; preapical area of inner margin of parameres concave; apex narrowly rounded, forming an acute angle pointing towards apex of aedeagus. Median lobe with lateral margins straight to somewhat sinuated, either parallel along basal 2/3 or converging from base; apex of aedeagus variable, wide to narrow, angulated to rounded; gonopore usually semicircular. Basal piece as long as 0.6-times the length of the median lobe, with lateral margins straight to sinuate; manubrium 0.3 times the length and clearly narrower than the basal piece at its base.

##### Variation.

There is variation in the shape and sharpness of the transverse ridge; the general shape of the aedeagus is consistent within the species, but varies in specific characters: shape of outer margins of parameres, width and shape of apex of parameres, shape of apex of aedeagus. There is one male specimen from Suriname (Collecting event SUR1F99 070; see Fig. 8H) in which the median lobe is longer than the parameres and the gonopore is rather oval.

##### Distribution.

Brazil (Amazonas), French Guiana, Guyana, Suriname, Venezuela (Bolívar). See Fig. 9C.

##### Biology.

Most specimens were collected in flight intercept traps. The species was also collected on the refuse pile of the ant *Acromyrmex hystrix*, under fermenting bark and by fogging a splintered tree trunk. *Q. similaris* has been collected at elevations between 25 and 350 m.

##### Remarks.

The male of *Q. similaris* was unknown until now. Specimens collected in Guiana were recognized as belonging to this species by the posteriorly markedly canaliculated pseudepipleura, a character shared with *Q. depressus*. There are no consistent external characters that distinguish both species, but the shape of the apex of the parameres is remarkably different.

### Key to the species of *Quadriops* Hansen, 1999

**Table:** 

1	Elytra with punctures randomly and uniformly distributed (see Fig. 1)	**2**
–	Elytra with punctures arranged as well defined longitudinal series, forming striae (see Figs 2, 3)	**3**
2	Elevation of mesoventrite as a narrow toothlike projection	***Q. dentatus* Hansen** (Fig. 1A–D)
–	Elevation of mesoventrite as a wide, transverse, straight blunt carina	***Q. acroreius* sp. n.** (Fig. 1E–H)
3	Serial elytral punctures simple; the ones on striae similar in size as those on the interstrial surface (Figs 6A, 3E–F)	***Q. clusia* sp. n.**
–	Serial elytral punctures ramified; the ones on striae evidently larger in size than those on the interstrial surface (Fig. 6B)	**4**
4	Surface of pseudepipleura smooth throughout; dorsal outline of body in lateral view moderately and uniformly convex	***Q. reticulatus* Hansen** (Fig. 3A–D)
–	Surface of pseudepipleura posteriorly markedly canaliculated (e.g. Fig. 2G), dorsal outline of body in lateral view nearly flat	**5**
5	Apex of parameres widely to narrowly rounded (Figs 7A–D)	***Q. depressus* Hansen** (Fig. 2A–D)
–	Apex of parameres angulate (Fig. 8)	***Q. similaris* Hansen** (Fig. 2E–G)

## Discussion

The data now strongly support the conclusion that *Quadriops* is an exclusively terrestrial genus. Previously, all but one specimen were from flight intercept traps, leaving their preferred habitat a mystery. One paratype specimen of *Q. reticulatus* was taken from oak forest litter, leading Hansen to consider them “apparently terrestrial”, though it should be noted that incidental collections of otherwise aquatic or semiaquatic taxa in leaf litter are not rare. We located older material that confirmed additional collecting events from sifted litter as well as rotten logs and sap flows, further substantiating the terrestrial habits of several species, including *Q. reticulatus* and *Q. similaris*. In 2013, AEZS first observed several *Quadriops clusia* specimens crawling on a rotting *Clusia* fruit in Guyana, and was able to subsequently collect several long series. Since that time, we have actively sought to collect in *Clusia* fruits on subsequent expeditions in Suriname and Guyana. In most of the cases in which we have collected in the fruits, we have been able to find at least one individual, and in some cases have encountered hundreds once again. We have collected hydrophilid larvae from these fruits as well, but we have not yet confirmed that they belong to *Quadriops* (other water scavenger beetles from the tribes Megasternini and Coelostomatini were also collected with *Quadriops* in the fruits). Interestingly, although several *Quadriops* geographically co-occur with *Q. clusia* in Guyana and Suriname, none have been found in *Clusia* fruits. Additionally, no *Quadriops* has been found in any aquatic or semiaquatic habitats despite extensive recent collecting activity in northern South America.

When *Quadriops* was first described from a total of 17 specimens by Hansen (1999), he discussed at relative length his difficulty in placing the taxon in a tribe before ultimately deciding on the (then subtribe) Acidocerina of the Hydrophilini (*sensu* Hansen 1991). These difficulties in placing *Quadriops* are three-fold: First, the tribe Hydrophilini as Hansen (1991) defined it at the time was in fact not monophyletic, with the acidocerines actually not closely related at all to the remaining groups of Hydrophilini (Short and Fikáček 2013). Second, he misinterpreted several characters: he described *Quadriop*s as having simple (non-bifid) mandibles when in fact they are clearly bifid in the species where the character was examined. Additionally, his assignment of which taxa had “systematic punctures”, an important character for grouping lineages within the family, was in part erroneous, leading him to code a number of taxa as lacking these punctures when in fact they possessed them (see Short and Fikáček 2013 for a discussion). Third, the derived terrestrial way of life of *Quadriops* has almost certainly been the cause for some of its more atypical morphologies such as reduced palps and lack of femoral pubescence. When these issues are taken into account, *Quadriops* is easily accommodated within the Acidocerinae (Short and Fikáček 2013).

## Supplementary Material

XML Treatment for Quadriops

XML Treatment for Quadriops acroreius

XML Treatment for Quadriops clusia

XML Treatment for Quadriops dentatus

XML Treatment for Quadriops depressus

XML Treatment for Quadriops reticulatus

XML Treatment for Quadriops similaris

## References

[B1] García M (2000) Una nueva especie de *Quadriops* Hansen, 1999 (Coleoptera: Hydrophilidae: Hydrophilinae) de Venezuela. Boletín del Centro de Investigaciones Biológicas 34(1): 59–65.

[B2] García M (2001) Nueva subtribu, género y especie de Hydrophilini (Coleoptera: Hydrophilidae) del extremo suroriental de Venezuela. Boletín del Centro de Investigaciones Biológicas Universidad del Zulia 35: 151–160.

[B3] Hansen M (1991) The hydrophiloid beetles. Phylogeny, classification and a revision of the genera (Coleoptera: Hydrophilidae). Biologiske Skrifter 40: 1–367.

[B4] Hansen M (1999) Fifteen new genera of Hydrophilidae (Coleoptera), with remarks on the generic classification of the family. Insect Systematics & Evolution 30(2): 121–172. https://doi.org/10.1163/187631200X00228

[B5] Komarek A (2004) Taxonomic revision of *Anacaena* Thomson, 1859. I. Afrotropical species (Coleoptera: Hydrophilidae). Koleopterologische Rundschau 74: 303–350.

[B6] Lawrence J Ślipiński A (2013) Australian Beetles: morphology, classification and keys. CSIRO Publishing, Melbourne-Australia, 576 pp.

[B7] Minoshima YN Komarek A Ôhara M (2015) A revision of *Megagraphydrus* Hansen (Coleoptera, Hydrophilidae): synonymization with *Agraphydrus* Regimbart and description of seven new species. Zootaxa 3930(1): 1–63. 25781810 10.11646/1–63

[B8] Short AEZ Fikáček M (2013) Molecular phylogeny, evolution and classification of the Hydrophilidae (Coleoptera). Systematic Entomology 38(4): 723–752. https://doi.org/10.1111/syen.12024

[B9] Short AEZ García M Girón JC (2017) Revision of the Neotropical water scavenger beetle genus *Globulosis* García, 2001 (Coleoptera: Hydrophilidae: Acidocerinae). Zootaxa 4232(2): 271–281. https://doi.org/10.11646/zootaxa.4232.2.10 10.11646/zootaxa.4232.2.10 28264397

[B10] Velázquez de Castro AJ (1998) Morphology and taxonomy of the genus *Sitona* Germar 1817, (I): The metendosternite (Col., Curc.). In: Colonnelli E Louw S Osella G (Eds) 2. Taxonomy, ecology and distribution of Curculionoidea (Coleoptera: Polyphaga). XX International Congress of Entomology, Florence (Italy), August 1996. Atti del Museo Regionale di Scienze Naturali, Torino, 109–123.

